# The information system stress, informatics competence and well-being of newly graduated and experienced nurses: a cross-sectional study

**DOI:** 10.1186/s12913-021-07132-6

**Published:** 2021-10-15

**Authors:** Anu-Marja Kaihlanen, Kia Gluschkoff, Elina Laukka, Tarja Heponiemi

**Affiliations:** Finnish Institute for Health and Welfare, P.O. Box 30, FI-00271 Helsinki, Finland

**Keywords:** Information systems, stress, nursing informatics, competence, well-being, newly graduated nurse

## Abstract

**Background:**

The use of information systems takes up a significant amount of nurses’ daily working time. Increased use of the systems requires nurses to have adequate competence in nursing informatics and is known to be a potential source of stress. However, little is known about the role of nursing informatics competence and stress related to information systems (SRIS) in the well-being of nurses. Moreover, the potential impact of nurses’ career stage on this matter is unknown. This study examined whether SRIS and nursing informatics competence are associated with stress and psychological distress in newly graduated nurses (NGNs) and experienced nurses.

**Methods:**

A cross-sectional study was conducted in Finland between October and December 2018. The participants were NGNs (*n* = 712) with less than two years of work experience and experienced nurses (*n* = 1226) with more than two years of work experience. The associations of nursing informatics and SRIS with nurses’ stress and psychological distress were analyzed with linear regression analysis. Analyses were conducted separately for NGNs and experienced nurses. Models were adjusted for age, gender, and work environment.

**Results:**

SRIS was associated with stress / psychological distress for both NGNs (β = 0.26 *p* < 0.001 / β = 0.22 *p* < 0.001) and experienced nurses (β = 0.21 *p* < 0.001/ β = 0.12 *p* < 0.001). Higher nursing informatics competence was associated with lower stress (β = 0.20 *p* < 0.001) and psychological distress (β = 0.16 *p* < 0.001) in NGNs, but not among experienced nurses.

**Conclusions:**

SRIS appears to be an equal source of stress and distress for nurses who are starting their careers and for more experienced nurses, who are also likely to be more experienced users of information systems. However, informatics competence played a more important role among NGNs and a lack of adequate competence seems to add to the strain that is already known to be high in the early stages of a career. It would be important for educational institutions to invest in nursing informatics so that new nurses entering the workforce have sufficient skills to work in increasingly digital health care.

**Supplementary Information:**

The online version contains supplementary material available at 10.1186/s12913-021-07132-6.

## Background

In recent years, the use of information technology has become an integral part of health care. Nurses, among other health care professionals, are required to adopt digital services and information systems as part of patient care [1, 2]. Information systems are expected to provide benefits from both economic and quality-of-care perspectives [3] but may also have unfavorable consequences for the end-users, such as increased stress and strain from learning and adapting information systems to the workflow [4–7].

Stress is considered to be a response to a stressful situation, and general measurement of stress symptoms has been widely used to measure well-being at work [8]. Well-being is also often viewed through psychological distress, which refers to a state of emotional suffering associated with demands and stressors that a person finds difficult to deal with in daily life (General Health Questionnaire, GHO, being one of the most widely used and established measurements) [9, 10]. Stress related to information systems (SRIS) refers to the stress caused by poorly functioning or constantly changing information systems [11]. Nurses’ SRIS has so far been little studied [12] compared with, for example, physicians’. Among physicians, SRIS has steadily increased in recent years [11], and using information systems with multiple functions has been associated with stress, especially if the work involves high time pressure [13–15]. Similarly, with nurses, information systems, such as demanding and detailed documentation in health records, have been found to take more time out of daily work than before [16, 17] and to be a considerable source of stress [18]. Studies have shown that nurses are burdened by the constant need to redefine their nursing expertise to provide care in digital environments [1]. Furthermore, the poor usability of the information systems has been associated with stress [19] and cognitive strain among nurses [20, 21].

Particularly the first years of professional nursing practice are known to be stressful with a lot of learning and adaptation [22, 23]. Therefore, the use of information systems may be particularly stressful for newly graduated nurses (NGNs). Challenges – such as managing heavy workloads, meeting working life expectations [24–26], and gaps in competence [25, 27, 28] – are found to be the main sources of stress for NGNs [22, 29]. In contrast, a higher level of task mastery has been shown to predict lower levels of stress [30].

Competence in nursing informatics, which refers to the processing of information and integrating information and communication technologies to promote the health of patients or clients [31, 32], has become a prerequisite for nurses’ performance in digitalizing health care [33–35]. NGNs entering working life are expected to be sufficiently competent in informatics and ready to use the information systems effectively [36]. However, it has been questioned how well current nursing curricula meet this competence need [37, 38]. Deficiencies have repeatedly been reported in students’ theoretical studies and in opportunities to practice using the systems and nursing informatics before starting working life [36, 39–41]. Best practices for teaching and ensuring sufficient informatics competence for nurses are also not clearly identified [39]. Moreover, nurses in different stages of their careers have expressed concerns about the lack of formal training and unrealistic competence expectations regarding information technology [1, 18].

Although the stress of NGNs, and nurses in general, is a widely studied topic [25, 42, 43], little is known about the potential impact of SRIS or nursing informatics competence on nurses’ overall well-being. Moreover, the potential impact of a nurse’s career stage on this matter is unknown. This study examined whether SRIS and nursing informatics competence are associated with stress and psychological distress in NGNs and experienced nurses.

## Methods

### Design

A cross-sectional survey study was conducted in Finland between November and December 2018.

### Participants and data collection

The study included two groups of participants. The first group consisted of NGNs (*n* = 6979) who had up to two years of work experience at the time of the data collection. This group included all nurses who graduated in Finland between 2016 and 2018. The second group consisted of registered nurses with more than two years of work experience (*n* = 10,000). These nurses were randomly picked from the Finnish Central Register of Valvira (the National Supervisory Authority for Welfare and Health). To obtain approximately equal numbers of respondents for both groups, the sample size of experienced nurses was defined to correspond to the number NGNs (taking into account possible non-response). Of these two groups, we were able to obtain email addresses for 3942 NGNs and 7000 experienced nurses from the register of the Finnish Association of Health and Social Care Professionals. Those whose email address was not obtained were excluded from the study (3037 NGNs and 3000 experienced nurses).

Nurses were invited to participate in the study and were sent a link to the electronic questionnaire via email. The invitation letter contained information on the purpose of the study, the voluntary nature of completing/sending the questionnaire, and the fact that the information will be processed both without identification of the participant and only by the members of the research team. A total of 712 NGNs (response rate: 18 %) and 1226 experienced nurses (response rate: 15 %) responded to the survey after three email reminders were sent.

### Measures

#### Stress

Stress was measured with a validated single-item measure of stress symptoms [8]: ‘*Stress means feeling tense, restless, nervous or anxious or being unable to sleep at night because one’s mind is troubled all the time. Do you feel stressed these days?*’ The item was answered on a five-point scale (ranging from 1 = ‘not at all’ to 5 = ‘very much’).

#### Psychological distress

Psychological distress was measured using four items (Cronbach’s alpha: α = 0.86) from the General Health Questionnaire (GHQ) [9, 44] that represent the anxiety/depression factor and is suggested to be the most preferable factor model for GHQ-12 [10]. Previously this measure has been associated with, for example, team climate and patient-related stress [45, 46]. Items, such as ‘*have you recently felt constantly under strain?’* were assessed on a four-point scale (ranging from 1 = ‘not at all’ to 4 = ‘a lot more than usually’).

#### SRIS

SRIS was measured by two items (α = 0.62) that assessed *how often a person has been distracted, worried, or stressed during the last six months about* (1) *constantly changing information systems and* (2) *difficult, poorly functioning IT equipment/software* [11] on a five-point scale (ranging from 1 = ‘very rarely or never’ to 5 = ‘very often or constantly’). The measure has been used in studies that have included physicians and has been associated with, for example, psychological distress (Heponiemi et al., 2018; Heponiemi et al., 2019).

#### Nursing informatics competence

Nursing informatics competence included four competence areas: (1) terminology-based documentation, (2) patient-related digital work, (3) general IT competency, and (4) electronic documentation according to structured national headings (Kinnunen et al., 2019). The participants were asked to evaluate *how well they have mastered the following competencies on a five-point scale (ranging from 1 = ‘very poorly’ to 5 = ‘very well’): documentation by using structured national headings* (competency 1); *supporting the patient to use electronic services* (competency 2); *basic IT skills* (e.g. data security information retrieval, word processing) (competency 3); and *electronic documentation of the patient care according to the nursing process* (competency 4) (α = 0.73).

Demographic information included age, gender, and the work environment (*emergency care, psychiatric and substance abuse services, specialized health care, elderly care, an outpatients department*, or *some other environment*).

The measures used in the study are presented in full in the supplementary material (Supplement 1).

### Data analysis

Multiple linear regression was used to examine the associations of SRIS and nursing informatics competence with stress and psychological distress. Analyses were conducted separately for both dependent variables (stress and psychological distress). First, as a preliminary analysis, we tested whether the possible associations of SRIS and informatics competence with stress and distress are different between NGNs and experienced nurses. This was done by combining the data from both nurse groups and included the interaction terms ‘SRIS*nurse group’ and ‘informatics competence*nurse group’ in the models predicting stress and distress. There was a significant interaction effect between nursing informatics competence and the nurse group for stress (*p* = 0.04) and psychological distress (*p* = 0.04). Therefore, the analyses were conducted separately for NGNs and experienced nurses. All models were adjusted for age, gender, and work environment. The analyses were conducted using R, version 1.2.1335.

## Results

The majority of the nurses were female. NGNs were on average 31 years old and experienced nurses 45 years old. In both groups, specialized health care and elderly care were the most common work environments. Nursing informatics competence was higher (*p* < 0.001) and SRIS was lower (*p* < 0.001) among NGNs compared with experienced nurses. The groups did not vary in the level of stress or psychological distress (see Table 1) 

**Table 1:** The characteristics of the participants and descriptive statistics

	Freq / % mean (SD)	
	**NGNs**	**ExNs**
Age	31.06 (8.73)	44.92 (11.18)
Gender		
Male	77 / 10.9 %	88 / 7.2 %
Female	627 / 89.1 %	1134 / 92.8 %
Work environment		
Emergency care	105 / 15.2 %	96 / 8.1 %
Psychiatric and substance abuse services	94 / 13.6 %	137 / 11.9 %
Specialised health care	260 / 37.7 %	376 / 31.8 %
Elderly care	161 / 23.4 %	306 / 25.9 %
Reception work	36 / 5.2 %	114 / 9.6 %
Other	33 / 4.8 %	154 / 13.0 %
Stress (range: 1–5)	2.72 (1.08)	2.71 (1.10)
Psychological distress (1–4)	2.09 (0.77)	2.10 (0.76)
Informatics competence (1–5)	4.02 (0.61)	3.86 (0.67)
SRIS (1–5)	2.57 (0.85)	2.94 (0.85)

NGNs = newly graduated nurses, ExNs = experienced nurses.

### The association of SRIS with stress and distress

**Fig. 1:**
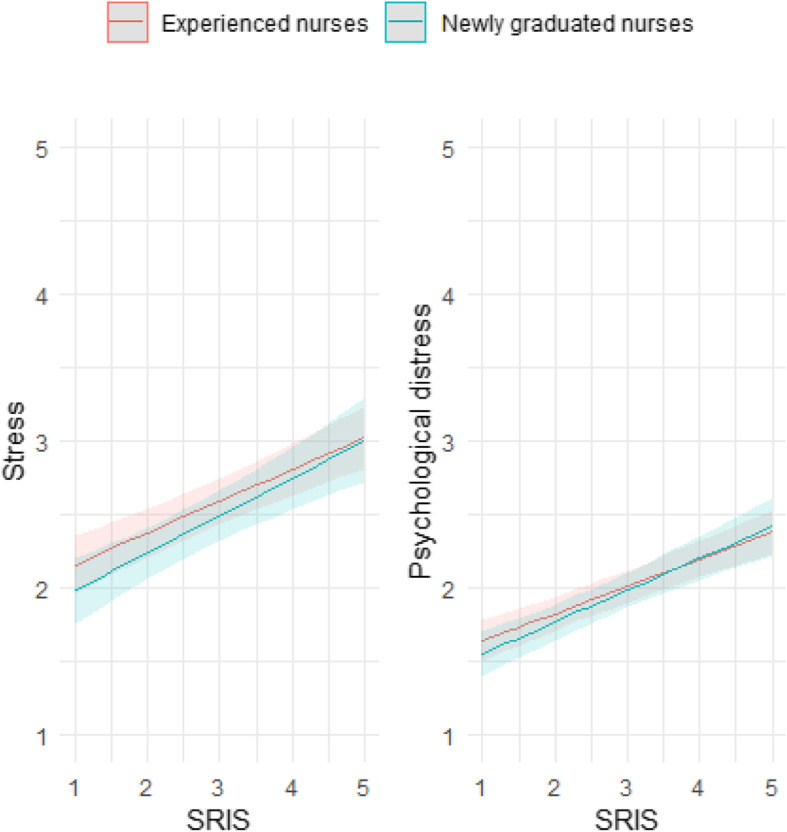
The association of SRIS with stress and distress in NGNs and experienced nurses (no interaction by nurse group)

SRIS was significantly associated with stress and distress in both NGNs and experienced nurses (see Table 2). The higher the nurses’ SRIS, the higher their stress and distress (see Fig. 1).

**Table 2:** The associations of independent variables with nurses’ stress and psychological distress

	NGNs		ExNs	
**Stress**	**Est.**	***p***	**Est.**	***p***
Informatics competence	-0.20	**< 0.001**	-0.02	0.65
SRIS	0.26	**< 0.001**	0.21	**< 0.001**
Age	-0.02	**< 0.001**	-0.02	**< 0.001**
Gender (female)	0.41	**< 0.001**	0.34	**0.01**
Work environment				
Emergency care	Ref	**-**	Ref	**-**
Psychiatric and substance abuse services	0.22	0.15	0.32	**0.03**
Specialised health care	0.21	0.08	0.13	0.31
Elderly care	0.29	**0.03**	0.20	0.12
Outpatients department	0.00	1.00	0.06	0.68
Other	0.09	0.69	0.17	0.24
**Psychological distress**				
Informatics competence	-0.16	**< 0.001**	-0.03	0.31
SRIS	0.22	**< 0.001**	0.12	**< 0.001**
Age	-0.01	**< 0.001**	-0.01	**< 0.001**
Gender (female)	0.18	0.06	0.17	**0.05**
Work environment				
Emergency care	Ref	-	Ref	**-**
Psychiatric and substance abuse services	0.24	**0.02**	0.11	0.28
Specialised health care	0.17	**0.05**	0.00	0.98
Elderly care	0.27	**0.00**	0.12	0.19
Outpatients department	0.12	0.40	-0.04	0.73
Other	0.29	0.06	0.05	0.47

NGNs = newly graduated nurses, ExNs = experienced nurses

### The association of nursing informatics competence with stress and psychological distress

**Fig. 2:**
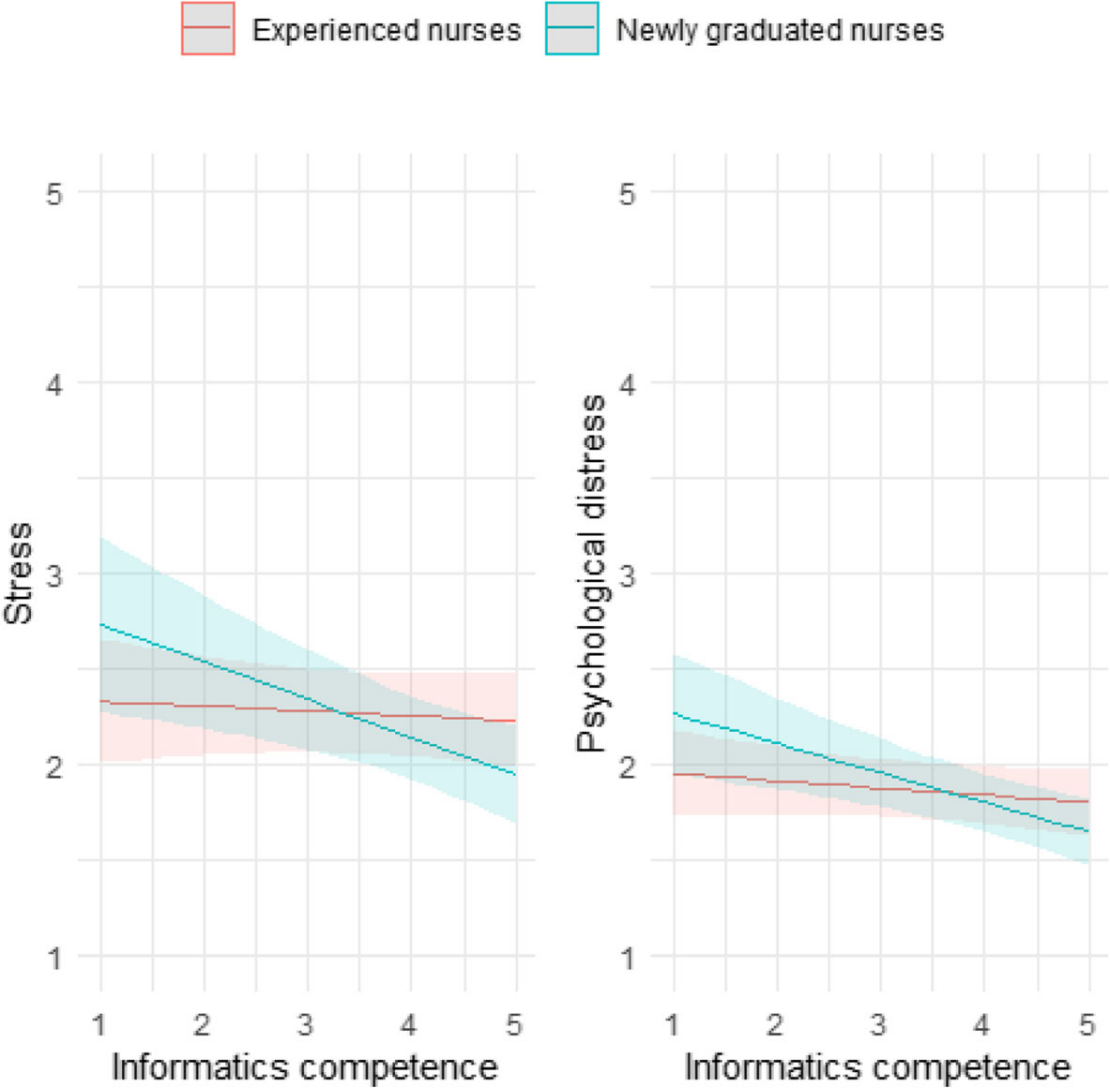
The association of nursing informatics competence with stress and distress in NGNs and experienced nurses (interaction by nurse group)

Nursing informatics competence was associated with stress and distress in NGNs but not in experienced nurses (see Table 2). Lower levels of competence were associated with high levels of stress and distress in NGNs, while in experienced nurses, competence was not associated with stress or distress (see Fig. 2.).

## Discussion

This study examined whether SRIS and nursing informatics competence are associated with stress and psychological distress in NGNs and experienced nurses. Our results showed that high SRIS was associated with high stress and distress in both groups. High nursing informatics competence, in turn, was associated with low levels of stress and distress in NGNs, but not among experienced nurses.

According to our results, SRIS appears to be a source of stress and psychological distress for nurses, regardless of the stage of their career. A previous study by Harris et al. (2018) has similarly found that stress due to electronic health record use may be associated with nurses’ burnout symptoms. Nurses are known to experience stress, especially in the implementation phase of new information systems [47], and this most likely occurs due to the high workload and lack of time that are typical barriers to the adoption and acceptance of the systems [48]. In our study, the SRIS levels were significantly higher for experienced nurses than for NGNs, but were still at a moderate level in both groups compared with, for example, the SRIS levels for Finnish physicians [14, 15]. Moreover, long experience with the use of information systems has been associated with lower SRIS levels in physicians, not the other way around [14]. The difference in nurses’ SRIS values can be partly explained by the fact that a significant proportion of NGNs represent a generation that is familiar with using a wide range of technologies, both in their studies and in their leisure time [49]. On the other hand, the widespread use of technology (e.g. in social interaction and knowledge sharing) does not directly indicate the ability to use these skills in academic or professional activities exists [41]. Nevertheless, NGNs attitudes and perceived self-efficacy in information and communication technology, and their understanding of its benefits appear to be higher than that of nurses with more experience and age [50, 51]. This may to some extent protect NGNs from SRIS in the workplace, but our results suggest that SRIS still affects their well-being.

Based on the results, a lack of sufficient nursing informatics competence may increase NGNs’ perceived stress and distress. NGNs evaluated themselves to be more competent in nursing informatics than experienced nurses, which is consistent with previous knowledge [34], although having more experience in the use of health information systems has previously been associated with higher informatics competence among nurses [33].

Integrating nursing informatics into nursing education has been a mission in many countries, but global and national differences in the use of technologies have made it difficult to determine best teaching practices and ensure the coherence of curricula [52]. This study confirms the need to invest in adequate informatics education as good competence may alleviate the stress and distress in the first years of a nurse’s work. Even though the level of informatics competence was not similarly associated with stress or distress among more experienced nurses, the provision of on-the-job training about informatics would also be extremely important. Previous studies have stated the need to develop, for example, nurses’ competence in using online services in patient care, supporting patients in utilizing these services [1, 53], structured documentation, and basic IT skills [54]. It is worth investing in the development of these competencies as there is repeated evidence of a link between the training received and nurses’ informatics competence [33, 55]. Moreover, the importance of nursing informatics for ensuring the quality of health care is well recognized internationally [56, 57].

So far, research knowledge on the stress caused by poorly functioning and constantly changing information systems is scarce among nurses, making it difficult to compare its potential effects on different health and social care settings and professionals at different career stages or to make international comparisons. As the use of various information systems is likely to increase globally in the future, further research is needed in this regard.

## Limitations

Certain limitations need to be considered when interpreting the results of this study. It should be taken into account that the study used the self-assessment of nursing informatics competencies and not, for example, objective tests that could have shown a different result for the nurses’ competence, and thus could have influenced the results. In addition, SRIS was measured with only two items with a Cronbach’s alpha value that was somewhat low, although still at an acceptable level [58]. The cross-sectional design must also be taken into account as it does not allow establishing a causal association of nursing informatics or SRIS with stress and distress. Although the analyses of the study controlled the age, gender, and work environment of the participants, we are also aware that there are potential confounding factors that may have influenced the associations. Finally, response rates in this study also remained relatively low, which may reduce the generalisability of results. Regarding the response rate, it is not possible to know the number of those who did not receive the email invitation, for example, due to an expired email account or spam filtering, which quite often happens with these email invitations. In addition to non-response, the exclusion of potential participants due to the lack of email addresses increases the possibility of selection bias.

## Conclusions

According to this study, poorly functioning, constantly changing information systems can be a substantial source of stress and psychological distress for NGNs as well as more experienced nurses. It is the responsibility of system vendors to further develop information systems to better support the work of healthcare professionals and not to increase their workload and strain. Our findings also suggest that good nursing informatics competence may be especially important for nurses who are starting their career in terms of preventing early career stress and distress, although adequate nursing informatics competence is known to be of the utmost importance and a prerequisite for performing work at any stage in a nurse’s career. It would be very important to take into account the stressors of nurses and invest in their well-being as this might mitigate nurses’ profession changes in a situation where there is already a global shortage of nurses. The tasks in nursing that require information management skills will increase in the future, thus, providing adequate and appropriate support and training in the use of information systems would be very important in both educational institutions and health care organizations.

## Supplementary information

**Additional file 1**

## Data Availability

The datasets used and analysed during the current study are available from the corresponding author on reasonable request.

## Abbreviations

NGN: Newly graduated nurse
SRIS: Stress related to information systems
