# Supplementary material for: The information system stress, informatics competence and well-being of newly graduated and experienced nurses: a cross-sectional study
Source: BMC Health Serv Res. 2021 Oct 15;21:1096. doi: 10.1186/s12913-021-07132-6 (PMC8518282; doi:10.1186/s12913-021-07132-6)
Supplement: Supplementary file 1 — Additional file 1 [file 12913_2021_7132_MOESM1_ESM.docx]

The information system stress, informatics competence and well-being of newly graduated and experienced nurses: A cross-sectional study

Anu-Marja Kaihlanen ^1^

[anu.kaihlanen@thl.fi](mailto:anu.kaihlanen@thl.fi)

Kia Gluschkoff ^1^

[kia.gluschkoff@thl.fi](mailto:kia.gluschkoff@thl.fi)

Elina Laukka^1^

[elina.laukka@oulu.fi](mailto:elina.laukka@oulu.fi)

Heponiemi, Tarja^1^

[tarja.heponiemi@thl.fi](mailto:tarja.heponiemi@thl.fi)

^1^ Finnish Institute for Health and Welfare

P.O. Box 30, FI-00271 Helsinki, Finland

Corresponding author:

Anu-Marja Kaihlanen

[anu.kaihlanen@thl.fi](mailto:anu.kaihlanen@thl.fi)

**Supplement 1**

**Measures used in the study**

**Stress:**

*Stress means feeling tense, restless, nervous or anxious or being unable to sleep at night because one’s mind is troubled all the time. Do you feel stressed these days?*

Response options:

1. Not at all

2. Only a little

3. To some extent

4. Rather much

5. Very much

***Psychological distress***

*The following questions concern your wellbeing within the last few weeks.*

1. Have you often remained awake lately because of your worries?
2. Have you felt unhappy or depressed lately?
3. Have you felt constantly overburdened lately?
4. Have you felt lately that you could not cope with difficulties?

Response options:

1. Not at all
2. No more than usually
3. Slightly more than usually
4. A lot more than usually

***Stress related to information systems (SRIS):***

*How often has each of the issues mentioned below clearly disturbed, worried or burdened you at work during the past 6 months?*

1. Constantly changing information systems

2. Difficult, poorly performing IT equipment/software

Response options:

1. Very rarely or never

2. Quite rarely

3. Every once in a while

4. Quite often

5. Very often or constantly

***Nursing informatics competence***

*How well do you feel you master the following competencies required by information systems?*

1. Documentation by using structured national headings

2. Supporting the patient to use electronic services

3. Basic IT skills (e.g. data security information retrieval, word processing)

4. Electronic documentation of the patient care according to the nursing process

Response options:

1. Very poorly

2. Quite poorly

3. Not poorly or well

4. Quite well

5. Very well

***Employment sector:***

*Principal occupations’ employment sector*

Response options:

1. Emergency care

2. Psychiatric and substance abuse services

3. Specialised health care

4. Elderly care

5. An outpatients department

6. Some other environment
